# Ballistic Response of a Glass Fiber Composite for Two Levels of Threat

**DOI:** 10.3390/polym15041039

**Published:** 2023-02-19

**Authors:** George Ghiocel Ojoc, Larisa Chiper Titire, Cristian Munteniță, Cătălin Pîrvu, Simona Sandu, Lorena Deleanu

**Affiliations:** 1 Faculty of Engineering, “Dunarea de Jos” University, 800008 Galati, Romania; 2 National Institute for Aero-Space Research (INCAS) “Elie Carafoli”, 061126 Bucharest, Romania; 3 Center for Research and Innovation for CBRN Defense and Ecology, 02512 Bucharest, Romania

**Keywords:** glass fiber composite, epoxy resin, ballistic impact, finite element model, cohesive zone model

## Abstract

This paper presents the behavior of composite panels based on glass fiber unidirectional fabrics and a bi-component epoxy resin under ballistic impacts that characterize two threat levels: FB2 and FB3, according to EN 1523:2004. The tested panels had characteristics kept in narrow ranges: thickness 18.26 ± 0.22 mm, mass ratio fabrics/panel 0.788 ± 0.015, surface density 27.51 ± 0.26 kg/m^2^. After testing the panels, the failure mechanisms of the panel were evidenced by scanning electron microscopy and photographs. Here the authors present a finite-element model at meso scale that was used for evaluating if the composite, initially tested at level FB2 (9 mm FMJ, v_0_ = 375 m/s), could withstand the higher level of impact, FB3 (projectile type 0.357 Magnum and impact velocity of v_0_ = 433 m/s). Simulation was performed in Explicit Dynamics (Ansys), keeping the same target but changing the projectile for the two different levels of threat. The results of the simulation were encouraging for making tests at level FB3, indicating the importance of alternating actual tests with simulations in order to achieve better protection with reduced surface weight. The simulation illustrated differences in impact duration and number of layers broken on the panel for each level. Validation of the model was based on the number of broken layers and the dimension of the delamination zone between the last two layers. Scanning electron microscopy was used for identifying failure mechanisms at the micro and meso scale. We found that damage to the composite was intensively dependent on impact velocity, this being quantitatively evaluated using the number of layers broken, the effect of delamination on separating layers and the deformation of the last layer.

## 1. Introduction

Composites with glass fiber fabrics, bonded with high-quality resins, are used for equipment protection, especially for vehicles and enclosed spaces that are exposed to projectiles [1,2,3,4,5,6,7]. The variables involved in the fabrication of such composites are numerous and their initial design requires dedicated documentation for, e.g., the quality of the glass fiber, types of fabrics, yarn and sublayer architecture, resin and technological aspects and thermal treatment [8,9,10].

Non-woven fabrics, also known as unidirectional fabrics, gained a market almost half a century ago as substitutes for woven or knitted textiles. Among their recognized advantages are lower price and rapid processing of composites [11], but in time, this type of fabric became evidentially adequate for particular applications especially, including ballistic protection systems [12,13]. Non-woven fabrics have, as base elements, high-quality fibers in terms of structure, length and mechanical characteristics. The yarns formed by these fibers are held together with auxiliary stitching yarns or/and very thin polymer foils that have a very low influence on the mechanical characteristics of the main fibers [8,12]. The fibers can be natural, synthetic (polymers) or from special resources (glass, carbon, ceramics), but polymeric, carbon and glass fibers are designated in designing components of high performance. Today, for ballistic protection, composites based on unidirectional fibers have become of interest, and research has intensified in particular for body armors and vehicles. There are numerous combinations of fabrics and resins and with the risk of the applications being high, tests are imperatively required on the final products. Of course, in order to limit the number of final tests, because they are expensive [14,15], research is generally performed on simplified samples and simulations using numerical solutions (as finite element method or smoothed-particle hydrodynamics method [16]).

Satkar A. R. et al. [17] reported the ballistic performances of a hybrid composite of three layers of glass fiber fabric and two layers of carbon fiber fabric in an epoxy matrix, based on a numerical simulation with ANSYS-Explicit Dynamic, for several impact velocities (400 m/s, 450 m/s and 500 m/s) and different fabric combinations. The results indicate that glass fiber fabric has better behavior but higher surface density.

Many reports deal with woven glass or polymeric fabrics for ballistic protection, but those for unidirectional or multiaxial fabrics are less common [18]. The importance of sub-layer architecture (meaning the layers and their orientations as prepreg, as supplied by the fabric producers) was pointed out in [19]; the prepreg formed using quadriaxial fabric (0°/90°/45°/−45°), namely LFT SB1plus, in a panel of 12 layers had a smaller BFS (back face signature [20]) compared to a panel of 24 layers of prepreg with biaxial fabric (0°/90°), namely LFT SB1, even if surface density and thickness were close.

The ballistic limit of E-glass/epoxy composite is higher than that of carbon/epoxy composite. The energy absorption in E-glass/epoxy composite is produced by fiber deformation and their tensile failures, whereas the energy absorption in carbon/epoxy composite includes less residual yarn deformation, but more shear plugging [21,22].

Ansari and Chakrabarti [18] present a numerical model for stratified layers of glass fibers, using anisotropic properties for the target, but the model was run without friction; therefore, it differs from reality. The coefficient of friction was set to 0.22 for textiles in [23] and Zhou Y. et al. [24] presented a model with friction (with the same friction coefficient COF = 0.22 between yarns), noting that the energy dissipated by friction was proportional to the impact velocity of the projectile for the studied range of velocities. Signetti S. et al. [25] used a model with the same friction law for the sliding contact between the projectile and the target layers, depending on relative sliding between nodes, taking into account both dynamic and static values. Ingles S. et al. [26] considered to have an optimum for the value of the friction coefficient in order to determine the maximum of the absorbed energy in a ballistic process when modeling fabrics with aramid fibers. Vescovini A. et al. [27] considered the same friction coefficient in a model with layers made of aramid fibers and glass fibers.

Mohan S. and Velu S. [28] proposed an analytical model to study the impact process of differently shaped projectiles penetrating into a unidirectional glass fiber reinforced cross ply with laminate with 8 layers and 7.1 mm thickness, but their experimental work evaluated the correspondence between model and test for low impact velocities (approx. 100 m/s), not for setting a protective design.

Ma D. et al. [29] presented a study with a model at meso scale for the panel (composed of layers with defined properties, based on testing the composite at different strain rates), having similar values for the strength limits of a layer and the same delamination model but with lower values. In 2022, Ma D. et al. [30] discussed several finite element models, at macro and meso levels, comparing the simulation results with the experimental ones. The models varied the mechanical characteristics as a function of strain rate and the accuracy of the residual velocity and damage aspects was improved by introducing the appropriate strain rate effect on the elasticity modulus and the strain at break.

Karthick P. and Ramajeyathilagam K. [31] published a numerical model for hybrid composites under impact (layers of fabrics with glass, carbon and aramid fibers, in hybrid sequences). To simulate the ballistic impact behavior of a glass fiber composite plate numerically, a MAT 54 enhanced composite damage material model was used, with Chang–Chang failure criteria, available in LS DYNA, but friction was not included.

Continuum and meso scale model response under 153 m/s velocity impact on a single woven layer was presented by Meyer C. S. et al. [32]. Their meso scale model included accurate fabric geometry and fiber volume fraction, rate-dependent matrix behavior, and important damage mechanisms including tow–tow delamination, tow pullout and frictional sliding. They selected μ = 0.5 for the friction coefficient in the delaminated zones and concluded that the meso scale model with appropriate values for mechanical characteristics depending on strain rate was closer to the experimental results, reporting the use of this model for a single threat.

This paper applies a relatively new design procedure [33] for armor development, studying ballistic performances of a composite made of quadriaxial glass fiber fabrics and high-quality epoxy resin. The first design solution was produced and tested in order to assess a threat level (FB2) and, based on these results and the literature, a finite element model at meso scale was elaborated, predicting that the solution could face a more dangerous impact (FB3), a conclusion supported by tests performed under requirements for this higher level of threat. The levels were those in the standard SR EN 1522:2004. Initially, a campaign of tests was performed for different thicknesses (number of layers of the same fabric) [34] for FB2, but the better response of the 24-layer composite to the FB2 threat suggested that it could be used for higher protection levels. Tests with projectile and parameters characterizing the FB3 level were performed and they indicate that the 24-layer composite is adequate for FB3 protection.

## 2. Materials and Methods

The composite had 24 layers of glass fiber fabric. The fabric was also layered into four unidirectional substrates (0°/+45°/90°/−45°), which gives the authors the opportunity to suggest that the fabric has quasi-isotropic behavior. The trade name is 1200 g/m^2^ quadriaxial glass cloth (0°/+45°/90°/−45°), with the code WTVQX1200-1 E-glass, Q1200E10Q [35,36]. Table 1 details the architecture of the fabric reinforcement.

**Figure 1:**
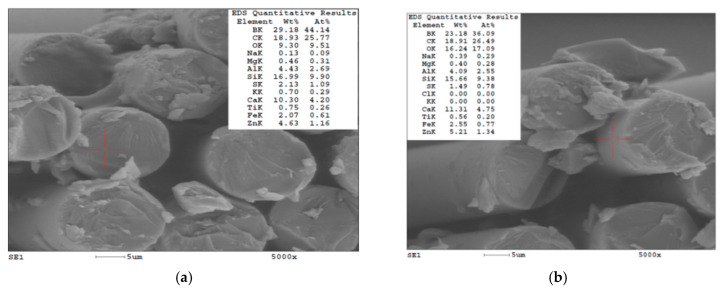
EDS elemental analysis of a glass fiber (red cross indicates the analyzed point). (**a**) Glass fiber core (cross section). (**b**) Glass fiber jacket.

Table 2 presents the average values of elemental composition for the core of the glass fibers (9 measurements on different fibers’ cross sections) and the jacket (5 measurements). Figure 1 shows the measurement points (indicated by a red cross) for two of these measurements. The differences in composition of the core and jacket of the glass fibers were small; thus, the fibers could be considered as having a homogenous composition as a whole body. For these fabrics, the boron content was higher than those attributed in the literature to high-resistance glass fibers of class D, E or S, which covers a range of 1% to 22%. This content should be attributed to the particular sourcing location of the raw materials. Boron decreases glass thermal expansion and not only increases resistance to vibration, strength, chemical resistance and long-life use, but also increases resistance to high temperatures and thermal shock.

After reviewing the documentation for resins used in fiberglass composites [21,37],, the two-component resin Biresin^®^ CR82 with hardener CH80-2 (Table 2) was selected from the products offered by the manufacturer Sika Group [38]. The mixing ratio must be followed accurately, as given in the resin data sheet, for optimal results. The final values for thermal and mechanical properties depend on the heat treatment after natural aging. The authors respected the producer’s heat treatment recommendations, as mentioned in [39], heating the panels with a rate of ca. 0.2 °C/minute, keeping the panels for 6 h at 60 °C (under the glass transition temperature and heat distortion temperature of fully cured neat resin) and then cooling them at a rate of ca. 0.5 °C/minute.

Biresin^®^ CR82 with hardener CH80-2, presented in Table 3, is a two-component epoxy resin for individual, manual lay-up, layer or vacuum forming and winding, especially for applications where a heat treatment temperature ≥75 °C cannot be applied. It can be used in marine and general composites for industry. The material and processing enclosure are recommended to have temperatures from 18 °C to 35 °C [39]. Using Biresin^®^ CH80-2 hardener, the composite can be removed from the mold at room temperature. Table 3 shows mechanical and thermal properties of the already formed and heat-treated resin, and it is observed that using the hardener CH80-2, a fairly high tensile limit was obtained.

Because of the panel dimensions (300 mm × 300 mm) and the individual pressing of each panel, the mixture of the two resin components was obtained in small quantities of 800 g of CR82 resin and 200 g of CH80-2 hardener for one mixture in order to keep it adequate to be laid up.

A layer of extraction wax was applied on the mold surface and the plywood sheets to ensure that the composite came off the mold more easily. CIREX CP 10 is an extraction wax (Airétec supplier) used for polyester and epoxy resins. The laying-up process consists of spreading the resin mixture on each layer with the help of a brush. The ballistic protective panels were kept in the press under load for at least 24 h.

Table 4 presents the characteristics of five fabricated panels of 24 layers, their average values and their standard deviations.

Considering the panel volume of 0.0016434 m^3^ and the volume of glass fiber fabrics as the ratio between the glass fiber fabric mass and an approximate density of 2300–2500 kg/m^3^ (as for glass fibers), a theoretical volume ratio of fibers/composite is 0.6–0.64, indicating the good quality of this composite. Key C. T. et al. [5] reported a 50–60% fiber volume fraction and 50–70% is also given in [37] due to statistical aspects of the processing of composites. Abdulmajeed A. A. et al. [40] reported a volume fraction of 51–57% for glass fiber for designed composites destinated for medical use.

**Figure 2:**
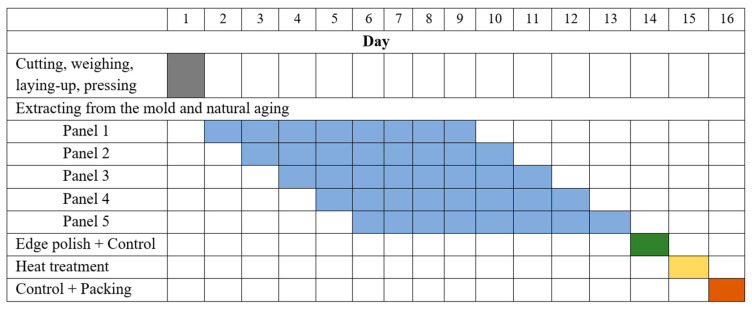
Gantt diagram for manufacturing a set of 5 panels made of 24 layers of glass fiber fabrics.

Figure 2 presents a Gantt diagram for manufacturing a set of five panels made of 24 layers of glass fiber fabrics, pointing out natural aging and the heat treatment. It is important to know the processing time for the final products.

Tests were performed at the Center for Research and Innovation for CBRN Defense and Ecology (Bucharest, Romania) and there the impact velocity was measured. Table 5 presents the characteristics of the levels FB2 and FB3. Each panel was hit in three points, with 120 mm between two points. The average measured values of the impact velocity were 375 m/s for FB2, meaning −6.25% of the average value in standard, and 433 m/s for FB3, meaning + 8.25% of the average value in standard. The impact velocity was noted as v_0_ and was measured using a projectile velocity measuring system, the Öehler 43 chronograph. The bullet hit from a distance of 5 m (normal conditions, [15]).

## 3. Results of the Simulation

### 3.1. The Model

Impact models are usually run at one of the following levels:

- Macro, with the target being one body, made of a single equivalent material, used especially for metallic shields [41];
- Meso, implying the layers as a continuous body with equivalent properties determined experimentally [27] and paying attention to modeling the bonding between them, being applied the cohesive zone model [42,43,44,45,46,47,48,49], as the designer wanted to have the thickness of the entire panel as small as possible; in this group, we can include fabrics modeled with yarns. This model is difficult to calibrate, taking into account the statistical response of such a multitude of bodies;
- Micro, when analyzing the behavior of a bundle of fibers under impact [50,51]; the big differences between dimensions of the fiber cross section and the projectile dimensions influence the model behavior.

**Figure 3:**
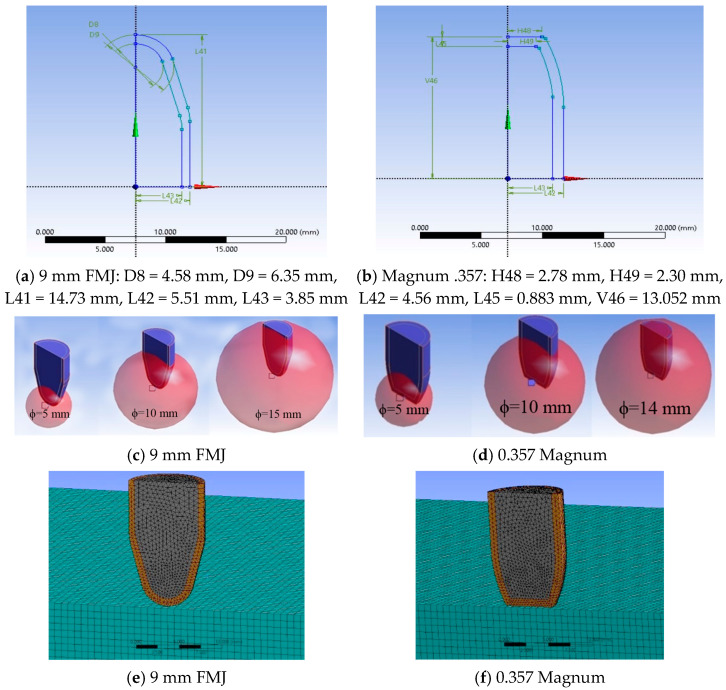
(**a**,**b**) Geometry of the projectiles, (**c**,**d**) influence spheres with graduate mesh elements for 9 mm FMJ and for 0.357 Magnum, (**e**,**f**) details of meshing for the projectiles and panel.

The modeled system contained the panel and the projectile. The panel was the same, but the projectile was different: one case used a 9 mm FMJ (full metal jacket) and the other case used a Magnum 0.357. Every bullet was made of two bodies with “perfect bonded” connection. The panel was composed of 24 layers. The element size and the discretization method are of importance, but in Ansys Explicit Dynamics the engineers must compromise between the element size, their number and the running time [52,53]. Figure 3a,b, presents the dimensions of the projectiles as parametric inputs and their meshing, after analyzing the drawings in [41,54,55,56].

For both projectiles, a tetrahedral network with at least two elements on the jacket thickness was used, obtained from an initial discretization, over which a mesh with three spheres of influence with increasing radii was added, the smallest sphere having the finest mesh (Figure 3), in order to have a relatively controlled growth of elements. For the first sphere, the element size was 0.35 mm, for the next sphere, it was 0.45 mm and for the largest one, it was 0.55 mm (Figure 3c,d).

The panel had a surface of 120 mm × 120 mm. The actual panel was 300 mm × 300 mm, allowing for three hits at a distance of 120 mm, forming an equilateral triangle, as required by the standard EN 1523:2004 Windows, doors, shutters and blinds. Bullet resistance. Test method. The simulation was run for only one hit on a smaller surface, but large enough to cause delamination.

The initial condition was the projectile velocity, considered here as v_0_ = 375 m/s for the 9 mm FMJ projectile and v_0_ = 433 m/s for the 0.357 Magnum projectile, these being the average measured values for the test campaign.

The 3D model was run as a half system, considering the symmetry plane of the system through the center of the squared panel and the longitudinal section of the projectile axis.

The limit conditions involved the lateral fixing of the panel. Each layer of the plate was embedded (fixed) on the lateral side surface of the layer.

For diminishing the running time, the bullet was very close to the panel, the distance between its tip and the panel being 0.258 mm.

The connection between layers was “bonded”, with “breakable’’ separating condition (the nodes being separated if certain values for the normal and shear stresses were overpassed, these being 100 MPa for traction and 70 MPa for shear, values characterizing the resin used for attaching the layers in panels).

### 3.2. Material Models of the Bodies Involved in the Model

In the run cases, the Johnson–Cook model was used for the core material (a lead alloy) and the jacket material (a brass alloy), based on the experimental data obtained by [57,58,59] (Table 6). Each layer of the panel had the mechanical characteristics in Table 6. Ou Y. et al. [1] reported high values of tensile strength limits for glass fiber yarn (around 1800 MPa) and values up to 800 MPa for the tested composite, depending on the strain rate, especially for passing from a lower (1/600 s^−1^) to a greater strain rate (158 s^−1^), and the resin had mechanical properties lower than those characterizing the resin model used in this study.

Based on the mentioned references and [60,61,62], the authors introduced a bilinear hardening model for the layer, here considered a homogenous and isotropic material, as different orientations of yarns on fabric sub-layers tend create properties in a narrower range [63,64].

Based on references [49,65], a cohesive zone model (CMZ) with zero thickness was introduced between the layers [53], the name in Explicit Dynamics commands for modeling the CZM being “Bilinear for interface delamination” and the failure criterion being set as “Fracture energies based debonding” for crack opening mode I. The parameters characterizing the cohesive zone model are given in Table 7.

### 3.3. Analysis of the Simulation Results

The first moment of the simulation was t = 7.5 × 10^−6^ s for both cases (one case for impact velocity v_0_ = 375 m/s with the 9 mm FMJ and the second case for v_0_ = 433 m/s with the 0.357 Magnum).

Due to the network meshing and the size of the elements, a slight asymmetry in layer rupture and equivalent stress distribution was possible, something that could also happen in an actual impact, as the material and geometry are not perfect. Thus, it was necessary to see where the rupture on each layer occurred and how the stress concentrators developed. The projectiles in both cases were allocated as transparent in order to better “see” the layer in the contact zone.

**Figure 4:**
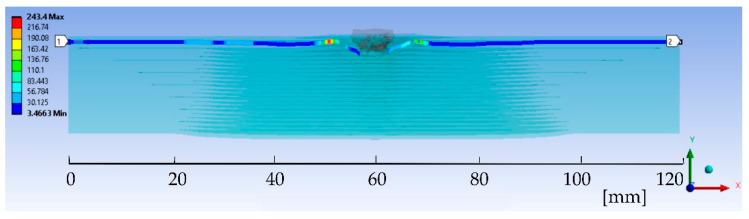
Detail of von Mises stress distribution for layer 2 and notations for analyzed length (stresses are given in MPa).

Figure 4 presents an example of von Mises stress distribution for the cross section of layer 2 at moment t = 1.5 × 10^−4^ s (the end of the simulation) indicating how the distance along the cross section of the panel is measured: 0 is at the left end of the layer, 60 mm is at the symmetry axis through the projectile and panel and 120 mm is at the right end of the layer.

**Figure 5:**
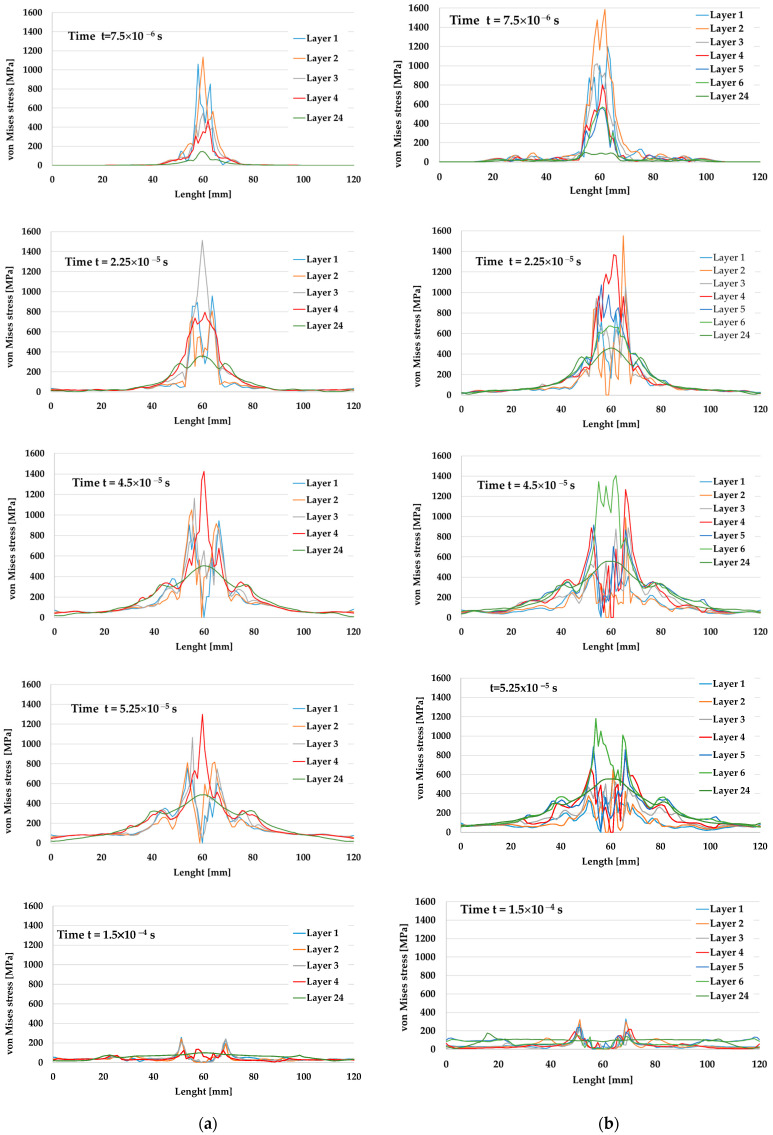
Von Mises stress distributions for broken layers and the last layer (the 24th layer) at different moments of the simulation. (**a**) 9 mm FMJ, v_0_ = 375 m/s. (**b**) 0.357 Magnum, v_0_ = 433 m/s.

Each graph in Figure 5 presents the von Mises stress distribution on the first layers involved in the failure process and the last layer. It was noticed that, at the end of the simulation, for v_0_ = 375 m/s, four layers were broken, and for v_0_ = 433 m/s, six layers were damaged. The plot succession shows at which moment each layer was broken (zero stress), the higher velocity being responsible for a more rapid and extended failure (in number of layers and affected volume in the target). Additionally, taking into account delamination, this failure process was more intense and enlarged on the analyzed cross length of the layers for the higher impact velocity. This is also evidenced by the photos in Figure 9b,d), where the delamination is visible as a lighter color compared to the general color of the panels.

At the first moment of the simulation, t = 7.5 × 10^−6^ s, the maximum values of von Mises stress were smaller for the 9 mm FMJ projectile (lower impact velocity of v_0_ = 375 m/s), but for the 0.357 Magnum, the maximum value was noticed on the second layer, close to the strength limit of the layer model, and there were stress values that announced possible delamination larger than that in the first case. The last layer was characterized by stress values under the layer yield limit for both cases, higher for the case with higher impact velocity. The load of the composite was more rapid and more intense for the impact with the 0.357 Magnum projectile. At t = 2.25 × 10^−5^ s, the second layer was broken (zero value for von Mises stress) under the 0.357 Magnum bullet, but the 9 mm FMJ one produced no break in the layers.

At t = 4.5 × 10^−5^ s, only two layers failed at impact velocity v_0_ = 375 m/s, but for v_0_ = 433 m/s, the first five layers were already broken. The 24th layer was stressed but under the yield limit.

The last moment of simulation, t = 1.5 *×* 10^−^^4^ s, evidenced low values of von Mises stress for all layers in both cases, lower than the yield limit of the layer material model.

**Figure 6:**
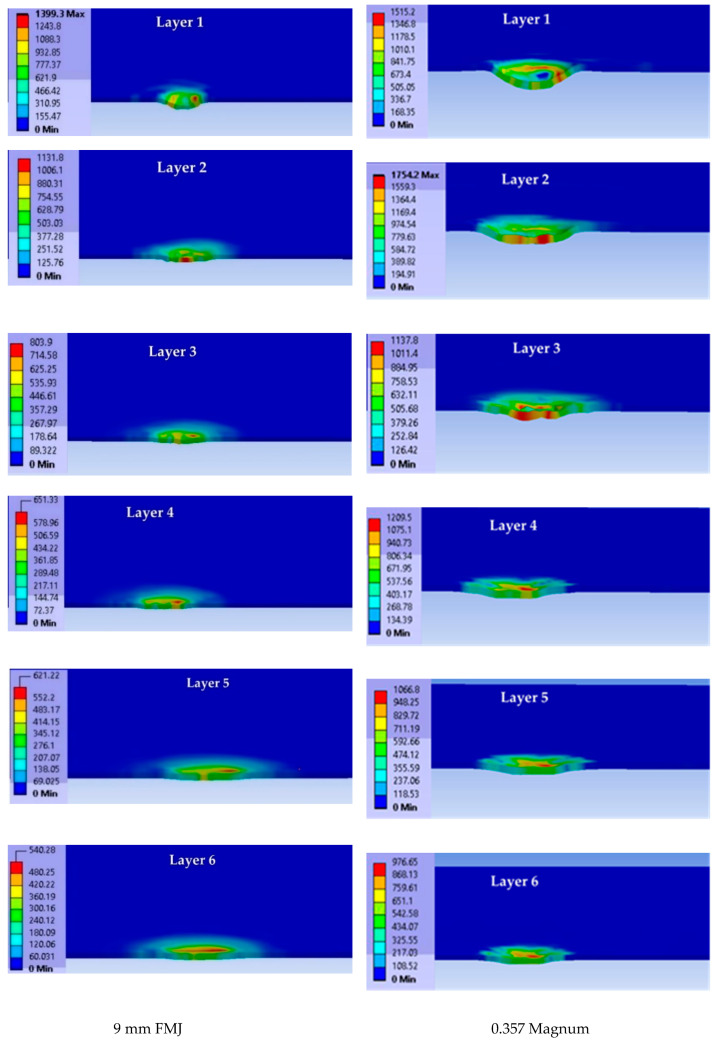
Images of each layer (layer 1 to layer 6) with von Mises stress distributions at moment t = 7.5 × 10^−6^ s (each image has its own color scale for stress values).

Figure 6 presents layer images in the first moment of the simulation (t = 7.5 × 10^−^^6^ s). The impact with the blunt projectile (0.357 Magnum) and higher velocity produced higher values of equivalent stress on each layer (only the first six layers are presented here). One may notice that the equivalent stress distributions have asymmetrical aspects, even if the model is based on symmetrical geometry. This is due to the meshing net and this happens in actual impact, too, as there are not perfect shapes and the actual materials also have structural and composition differences that could generate asymmetry in material loading. 

**Figure 7:**
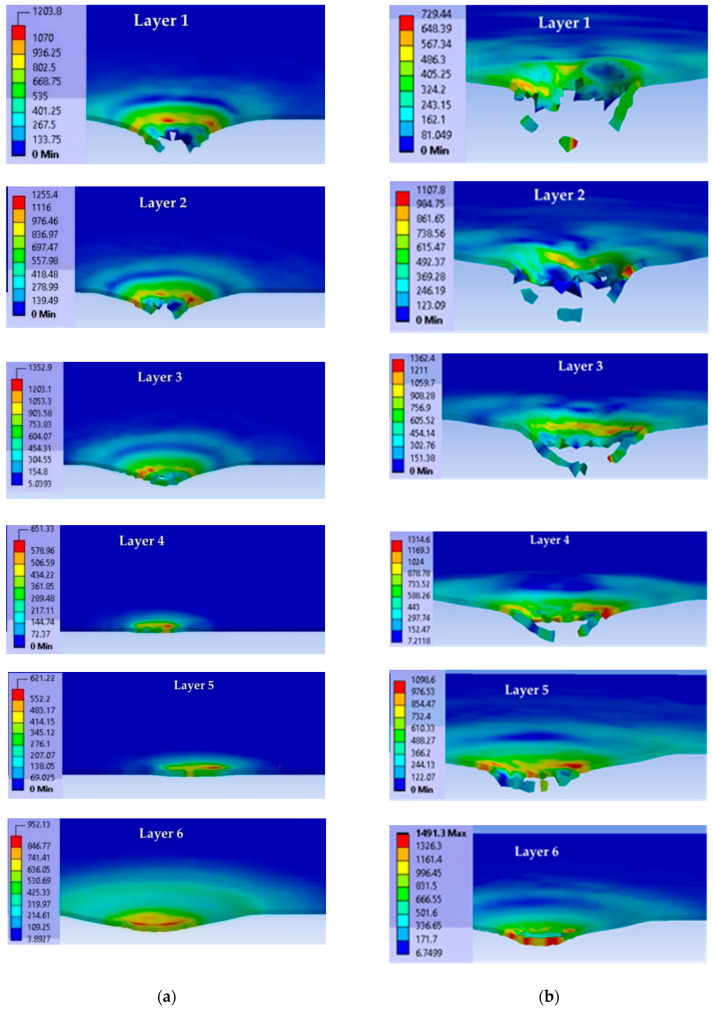
Cross section of each layer of the panel (the first six layers of each case) at the time moment t = 4.5 × 10^−5^ s (projectile is set as transparent). (**a**) For impact with 9 mm FMJ. (**b**) For impact with 0.357 Magnum.

This analysis should be correlated with the images obtained at the same moments, as the break of a certain layer could be initiated in a zone not included in the analyzed path. See Figure 7, layer 6 of the panel hit by the 0.357 Magnum bullet.

For the impact with the 9 mm FMJ projectile, the affected (stressed) zone had lower values for each layer compared to the impact produced by the other projectile. At t = 7.5 × 10^−6^ s, the biggest difference between the maximum values of von Mises stress was found on layer 4 (almost 50%). For the impact with the 0.357 Magnum projectile, layer 2 was the most stressed and layer 1 had a semi-circular zone of high stress, the other layers having small concentrated zones.

Figure 7 presents von Mises stress distributions on each layer at the moment t = 4.5 × 10^−5^ s for impact with the 9 mm FMJ at v_0_ = 375 m/s (left column) and impact with the 0.357 Magnum at v_0_ = 433 m/s (right column).

After this moment, there were no broken layers. The layer fragmentation was more intense for the higher impact velocity and these small fragments could be seen in SEM images on the last not-broken layer (see Figure 12a,d).

**Figure 8:**
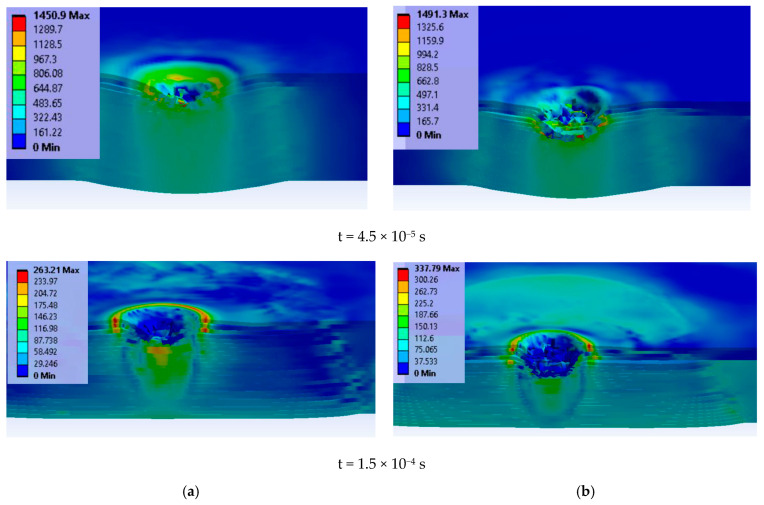
Cross section of the panel, at different time moments, for each modeled case (projectile is transparent) (color scale is given for each image, in MPa). (**a**) 9 mm FMJ. (**b**) 0.357 Magnum.

Figure 8 presents a cross section in the panel for two moments: at t = 4.5 × 10^−5^ s, there are similar local high values of von Mises stress, but delamination is more pronounced and the deformation on the last layer is greater for the higher impact velocity; at the last moment of the simulation, t = 1.5 × 10^−4^ s, the peripheries of delamination are well differentiated.

Comparing the images of the panels at t = 4.5 × 10^−5^ s to those at t = 1.5 × 10^−4^ s (the simulation’s end), one may notice that even if there were no more breaks in the layers until the end of simulation, the delamination continued. Small fragments, detached from superior failed layers, embedded (pressed) into the following layers. At this final moment, values for von Mises stress were lower than the yield limit of the glass fiber yarns and anterior delamination allowed for the bending of the layers around the direct impact zone.

The authors selected as the criterion for model validation the number of broken layers, and Table 8 presents this characteristic for both cases, the model and the actual tests.

## 4. Analysis of the Failure Mechanisms after Actual Tests

Failure mechanisms should be discussed for each scale level of the target [66]:

- Micro, including glass fibers and resin damage;
- Meso, here including delamination and failure of the projectile;
- Macro, including qualification of the composite and partial or total penetration, evidenced by photos taken of the entire panel or large areas or sections of it.

**Figure 9:**
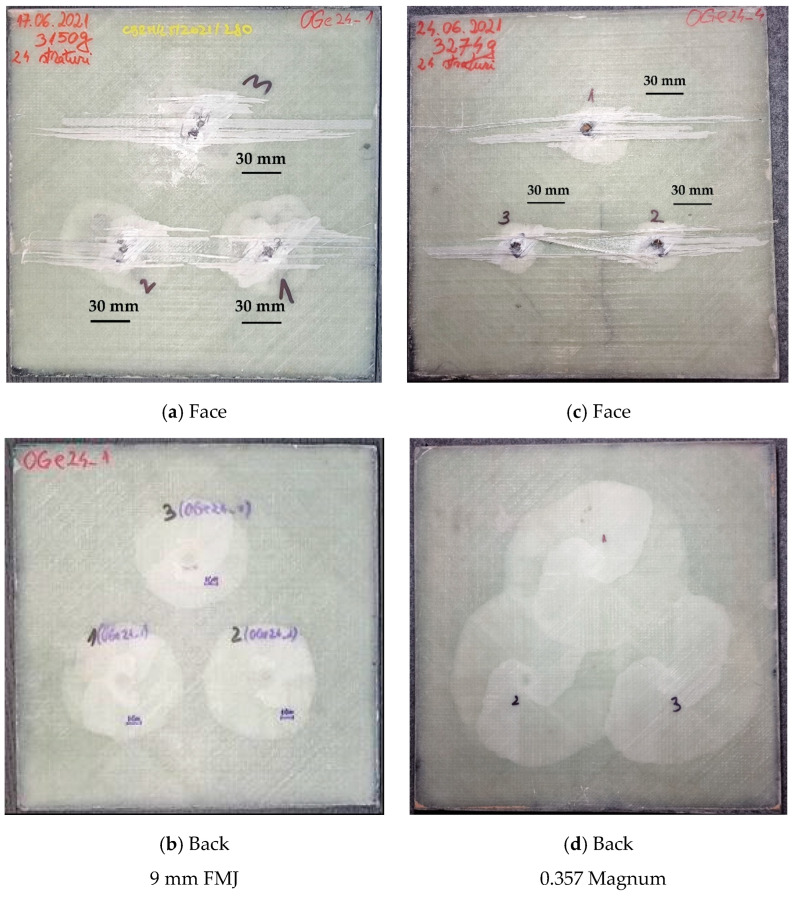
Aspects of the panels after test.

Figure 9 presents macro photos of the panels. The projectiles’ arrests have similar aspects all the three fires. It means that the panel is able to endure multiple hits at a close vicinity (the distance between fires was 120 mm).

A delamination of several yarns on the first layer of glass fibers that does not affect the integrity of the panel is visible. Delamination is visible on the panel back, with smaller circular zones for the lower impact velocity and overlapping, almost circular zones for the other impact velocity. The shape and color tones of the delamination suggest a process that advanced unevenly between layers. This is more visible in a cross section of the panel, obtained via high-speed cuts in dry conditions.

**Figure 10:**
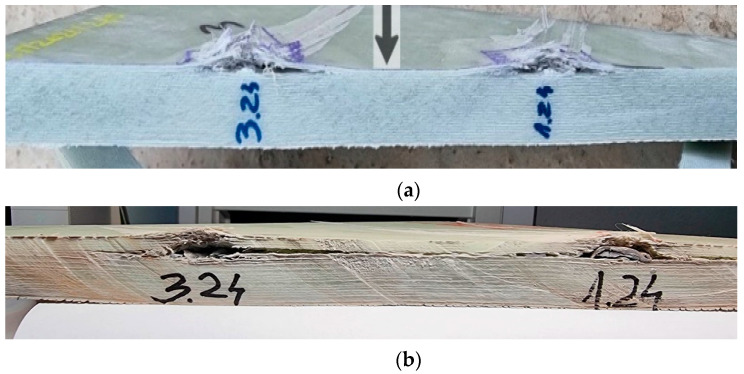
Cross-sections of lines with two fires: fire 1 (1.24) and fire 3 (3.24) for (**a**) 9 mm FMJ, fire 1 (coded 1.24) and fire 3 (coded 3.24) and (**b**) 0.357 Magnum fire 1 (coded 1.24) and fire 3 (coded 3.24).

**Figure 11:**
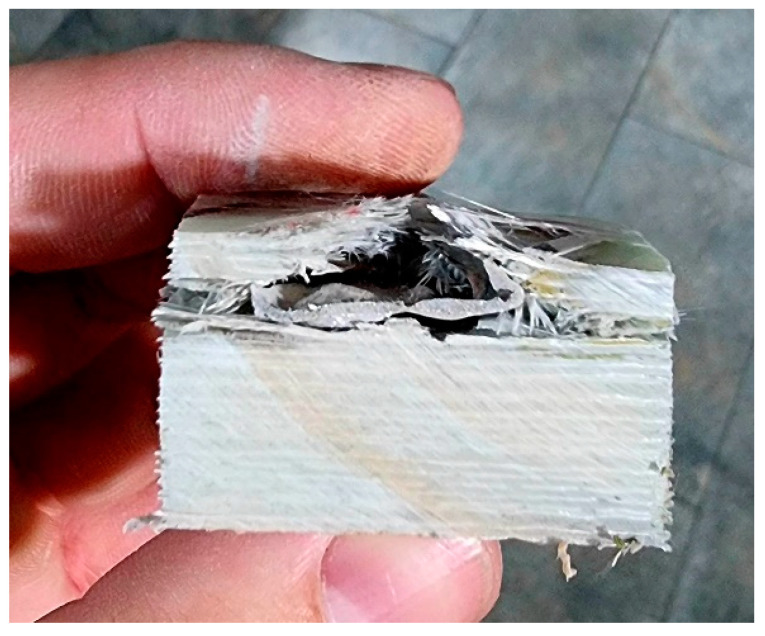
The cut sample for SEM investigation (after test with 0.357 Magnum).

The characteristics of the cross-sections indicate the following (Figure 10): the projectile with lower impact velocity and round head was flattened, but also rebounded to raise up the first broken layers (Figure 10a); meanwhile, the pointed projectile with higher impact velocity (Figure 10b) penetrated more layers and the raising of the broken layers was smaller, but the delamination was larger and very visible on many layers beneath the layers that stopped the projectile. Figure 11 presents how the penetration volume was cut in order to be examined with use of the scanning electron microscope. The surfaces of interest (the impacted face and the cross section) were gold-coated to obtain high-quality images.

**Figure 12:**
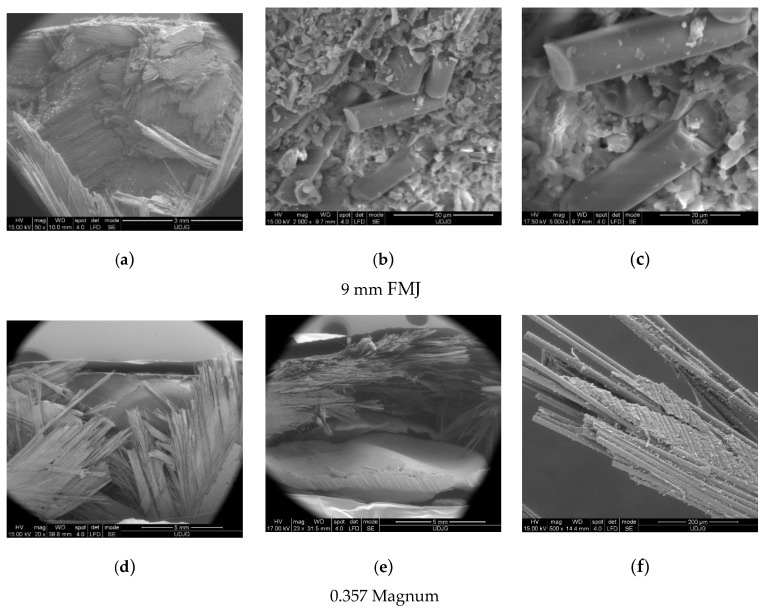
SEM images of the panel cross-section: (**a**–**c**) for 9 mm FMJ, (**d**–**f**) for 0.357 Magnum.

Figure 12 shows SEM images of the panel after impact with the 9 mm FMJ (**a**–**c**) and the 0.357 Magnum (**d**–**f**):

(a) Top view of the penetration hole produced by the 9 mm FMJ;
(b) Detail of the surface that the projectile was stopped on (fragments of the projectile are not in this image, but there are glass fiber fragments);
(c) Shear cut of a glass fiber (up), typically for impacted glass fibers, meaning a cut surface almost perpendicular to the fiber axis;
(d) Top view of the penetration hole produced by the 0.357 Magnum. On the bottom the flattened projectile is visible, less fragmented compared to the 9 mm FMJ;
(e) Detail in cross section, with flattened projectile and hole, not very cylindrical due to the different orientation of the yarns in each sub-layer;
(f) Detail of cut fibers and the aspect of delamination, revealing the detaching of the fibers of different orientation and other fibers remaining in the matrix.

Nunes S. G. et al. [67] presented experimental results on panels made of aramid fibers with similar projectiles for an impact velocity of 430 m/s (0.357 Magnum), obtaining zero residual velocity with an area density of 17.61 kg/m^2^ but no “reserve” on the panel thickness, meaning that the last layers were damaged; their model, with many similarities to this one, presented a virtual residual velocity of 195 m/s. Adding 25–30% of the aramid fabric thickness of 14.5 mm, this became 18.125–18.85 mm. Additionally, in this study, the BFS (back face signature), which is a request for body armor [20], was not given, as this composite is destinated for vehicle or equipment protection.

For the glass fiber panel (with 24 layers of quadriaxial fabrics) of 24.15 mm hit by the same projectile, the unbroken layers numbered 19, meaning too much “reserve”; this means that the range of thickness or the number of layers should be investigated for panels with less than 24 layers in order to optimize this panel. It could also be tested (virtually first) at a higher level.

## 5. Conclusions

This numerical and experimental study shows that alternating simulation of an impact at meso scale (target made of layers) using a finite element method and actual tests could be advantageous for designing ballistic protective composites, as elaborated by the authors, based on quadriaxial glass fiber fabrics and epoxy resin. Analyzing the behavior of the composite against a projectile 9 mm FMJ (v_0_ = 375 m/s), in actual and virtual tests, the finite element model presumed that the same composite would behave reliably for another threat (0.357 Magnum, v_0_ = 433 m/s). Tests performed for this level confirmed that the manufactured composite endured this threat as well. This designing method for systems with high risk may be used successfully for impact-protective systems.

The damage to a composite is intensively dependent on impact velocity and projectile type (shape, materials and mass), this being quantitatively evaluable by the number of broken layers, the layers’ delamination and the deformation of the last layer.

Simulation allows for virtually separating the impact stages on such a composite as well as evaluating the response of the same composite under different threat levels, based on already performed tests for only one threat, in order to optimize the design of the panel (especially diminishing surface density while keeping the same safety requirements) or recommend the same system for facing a higher threat level. The simulation results, validated by initial tests, are useful for reducing test costs, optimizing a certain protective system by diminishing its surface density or thickness while fulfilling the safety requirements, or recommending the system for a higher level of protection (as was the case in this study). Of course, the acceptance of a composite system for a higher level of protection requires actual standardized tests.

This study presents the behavior of the same panel—a composite based on quadriaxial glass fiber fabrics and an epoxy resin—under ballistic impacts that characterized two threat levels: FB2 and FB3, according to EN 1523:2004. We presented a model at meso scale that was used for evaluating if the composite, initially tested at level FB2 (9 mm FMJ, v_0_ = 375 m/s) could face a superior level of impact, FB3 (0.357 Magnum and v_0_ = 433 m/s). The simulation was performed using Explicit Dynamics (Ansys), keeping the same target but changing the projectile as required for the two different levels of threat. The results of the simulation are encouraging for testing at level FB3, indicating the importance of alternating actual tests with simulations in order to obtain better protection with reduced surface weight. The simulation indicated differences in impact duration and number of layers broken in the panel for each tested level. Validation of the model was performed for the FB2 test based on the number of broken layers and the dimension of the delamination zone between the last layers. Scanning electron microscopy was used for identifying the failure mechanisms at micro scale.

## Figures and Tables

**Table 1:** Architecture of the glass fiber fabric [35,36].

Layer	Yarn Orientation	Fiber Type	Area Weight
1.	0°	600 Tex	283 g/m^2^
2.	45°	300 + 600 Tex	300 g/m^2^
3.	90°	600 Tex	307 g/m^2^
4.	−45°	300 + 600 Tex	300 g/m^2^
	Stitch	76 Dtex	10 g/m^2^
Total surface weight: 1200 g/m^2^ (±%3)			

**Table 2:** Elemental analysis (EDS) of the glass fibers [34].

**Element Average (wt. %) (9 Measurement on Cross Section of Fibers on the Same Yarn)**													
**B**	**C**	**O**	**Na**	**Mg**	**Al**	**Si**	**S**	**Cl**	**K**	**Ca**	**Ti**	**Fe**	**Zn**
29.3	24.7	9.0	0.3	0.4	3.5	14.2	1.7	0.2	0.2	8.8	0.5	2.1	4.4
**Element Average (Wt. %) (4 Measurement on External Surface of Fibers on the Same Yarn)**													
30.8	24.6	9.7	0.5	0.6	3.1	10.6	1.5	0.2	0.5	7.8	1.0	2.8	5.6

**Table 3:** Characteristics of resin Biresin^®^ CR82 and hardener Biresin^®^ CH80-2 [39].

**Characteristics**		**Resin (A)** **Biresin^®^ CR82**	**Hardener (B)** **Biresin^®^ CH80-2**
Mixing ratio, parts by weight		100	27
Viscosity at 25 °C, mPa·s		~1.600	~80
Density at 25 °C, g/mL		1.11	0.99
**Mixture**			
Potlife, 100 g/RT (approx.), minutes		~80	
Mix viscosity, 25 °C (approx.), mPa·s		800	
**Characteristics**	**Tested According to**	**Units**	**Resin Biresin^®^ CR82 (A) with hardener CH80-2**
Tensile strength	ISO 527	MPa	85
Tensile elasticity modulus	ISO 527	MPa	3250
Elongation at break	ISO 527	%	5.0
Flexural strength	ISO 178	MPa	125
Flexural E-Modulus	ISO 178	MPa	3200
Compressive strength	ISO 604	MPa	107
Density	ISO 1183	g/cm^3^	1.16
Shore hardness	ISO 868	-	D 84
Impact resistance	ISO 179	kJ/m^2^	21
Typical thermal properties of fully cured neat resin			
Heat distortion temperature	ISO 75A	°C	77
Glass transition temperature	ISO 11357	°C	89

**Table 4:** Characteristics of the panels with 24 layers of quadriaxial glass fiber fabric.

Panel	Fabrics Mass	Panel Mass	Resin Mass *	Mass Ratio Fabrics/Panel **	Surface Density ***	Thickness in 4 Points				
						1	2	3	4	Average
	[g]	[g]	[g]		[kg/m^2^]	[mm]				
0	1	2	3	4	5	6	7	8	9	10
1	2500	3273	773	0.763	27.77	18.37	18.46	18.60	18.12	18.38
2	2510	3118	608	0.805	27.88	17.21	18.53	17.37	18.23	17.83
3	2460	3150	690	0.781	27.33	18.61	18.04	18.96	18.20	18.45
4	2460	3174	714	0.775	27.33	18.24	18.02	18.76	17.97	18.24
5	2450	3200	750	0.765	27.22	18.53	18.23	18.46	18.37	18.39
Average	2476	3183	707	0.778	27.51					18.26
Max	2510	3273	773	0.805	27.88					
Min	2450	3118	608	0.763	27.22					
Standard deviation	24.17	52.51	57.17	0.015	0.266					0.225

*: Resin mass = Panel mass—fabrics’ mass (column 2—column 1), **: Mass ratio fabrics/panel = fabrics’ mass/panel mass (column 1/column 2), ***: Surface density = Panel mass/panel area (=0.09 m^2^).

**Table 5:** Characteristics of levels FB2 and FB3 as given in the classification and requirements for testing with hand guns and rifles [15].

Class	Type of Weapon	Caliber	Bullet		Test Condition	
			Type	Mass [g]	Test Range [m]	Bullet Velocity [m/s]
FB2	Hand gun	9 mm Luger	FJ/RN/SC	8.0 ± 0.1	5 ± 0.5	400 ± 10
FB3	Hand gun	0.357 Magnum	FJ/RN/SC	10.2 ± 0.1	5 ± 0.5	430 ± 10
FJ—full metal jacket, RN—round-nose bullet, SC—soft core (lead).						

**Table 6:** Characteristics of materials involved in this model.

Property	Jacket (Brass)	Core (Lead Alloy)
Density [kg/m^3^]	8450 *	11350 *
Specific heat at constant pressure [mJ/(kg °C)]	380	1.288 × 10^5^
Young modulus [MPa]	90,000 * (115,000, [59])	16,000 [59]
Poisson coefficient	0.344	0.44
Temperature [°C]	22	22
Constants for Johnson–Cook model		
Initial yield limit [MPa]	90 [58] (80 [59])	1 [59] (0, [58])
Hardening constant [MPa]	628 [58]	55 [58]
Hardening exponent	0.72 [58]	9.8 × 10^−2^ [58]
Constant for strain rate	0.266 [58]	0.231 [58]
Thermal softening exponent	1 [58]	1 [58]
Quasi-static strain rate threshold (s^−1^)	604 [59]	221 [59]
Melting temperature [°C]	927 *	327.5 *
Equivalent plastic strain at break (EPS)	0.75 *	0.75 *
Mechanical properties of a layer		
Property	Value	
Density [kg/m^3^]	1904 *	
Specific heat at constant pressure [mJ/(kg °C)]	6 × 10^5^ *	
Young modulus [MPa]	50,000 [60]	
Poisson coefficient	0.3065 *	
Temperature [°C]	22	
Isotropic bilinear hardening model		
Initial yield limit [MPa]	550 * (577 for 495 s^−1^, in [60])	
Tangent modulus [MPa]	10,000 *	
Temperature [°C]	22	
Equivalent plastic strain at break (EPS)	0.12 *	

*: Selected by the authors. Values in brackets are from the indicated reference.

**Table 7:** Parameters for modeling the bilinear strength and energy at break in interlaminar delamination (values selected by the authors).

**Parameters for Modeling the Bilinear Strength in Interlaminar Delamination**					
Tempe-rature, °C	Maximum normal traction stress at the interface, MPa	Normal displacement jump at completion of debonding, mm	Maximum tangential traction stress at the interface, MPa	Tangential displacement jump at completion of debonding, mm	Ratio
22	70	5	50	0.1	0.3
**Parameters for Energy at Break in Interlaminar Delamination**					
Tempe-rature, °C	Maximum normal contact stress, MPa	Critical fracture energy for normal separation, J/m^2^	Maximum equivalent tangential contact stress, MPa	Critical fracture energy for tangential slip, J/m^2^	Artificial damping coefficient, s
22	100	3000 [65]	-	-	0.1

**Table 8:** Number of broken layers for each case.

Case (Projectile)	Number of Broken Layers	
	Experimental	Numerical
9 mm FMJ	2–3	3
0.357 Magnum	5	5

## Data Availability

All data having references could be publicly accessed.
